# Tetra-μ-acetato-κ^8^ *O*:*O*′-bis­{[*N*-(4-chloro­phen­yl)-4-methyl­pyridin-2-amine-κ*N* ^1^]copper(II)}

**DOI:** 10.1107/S1600536810030187

**Published:** 2010-08-04

**Authors:** Zainal A. Fairuz, Zaharah Aiyub, Zanariah Abdullah, Seik Weng Ng, Edward R. T. Tiekink

**Affiliations:** a Department of Chemistry, University of Malaya, 50603 Kuala Lumpur, Malaysia

## Abstract

In the crystal structure of the title complex, [Cu_2_(CH_3_COO)_4_(C_12_H_11_ClN_2_)_2_], the complete binuclear mol­ecule is generated by a crystallographic centre of inversion; the four acetate groups each bridge a pair of Cu^II^ atoms. The coordination of the metal atom is distorted octa­hedral within a donor set defined by four O atoms, the heterocyclic N atom and the second Cu atom. The pyridine ring is twisted with respect to the benzene ring, forming a dihedral angle of 33.9 (2)°. An intra­molecular N—H⋯O hydrogen bond is present between the amino group and a carboxyl O atom. Inter­molecular inter­actions of the C—H⋯π type link mol­ecules in the crystal structure.

## Related literature

For examples of tetra­kis­acetato­bis­[(substituted 2-amino­pyrid­yl)copper] complexes, see: Barquín *et al.* (2004 ▶); Seco *et al.* (2004 ▶); Sieroń (2004 ▶); Fairuz *et al.* (2009 ▶).
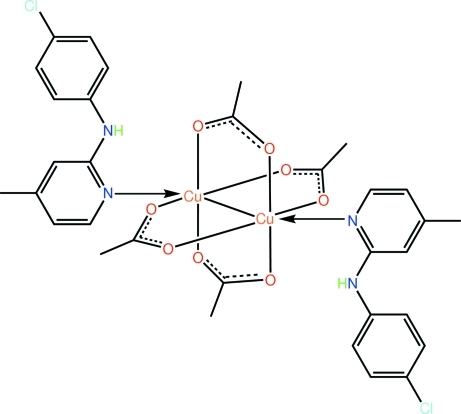


## Experimental

### 

#### Crystal data

[Cu_2_(C_2_H_3_O_2_)_4_(C_12_H_11_ClN_2_)_2_]
*M* *_r_* = 800.61
Monoclinic, 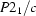

*a* = 11.7430 (17) Å
*b* = 15.619 (2) Å
*c* = 9.9866 (14) Å
β = 109.901 (2)°
*V* = 1722.3 (4) Å^3^
*Z* = 2
Mo *K*α radiation
μ = 1.45 mm^−1^
*T* = 296 K
0.35 × 0.25 × 0.05 mm

#### Data collection

Bruker SMART APEX CCD diffractometer
Absorption correction: multi-scan (*SADABS*; Sheldrick, 1996 ▶) *T*_min_ = 0.632, *T*_max_ = 0.931
11432 measured reflections
3951 independent reflections
2460 reflections with *I* > 2σ(*I*)
*R*_int_ = 0.065

#### Refinement

*R*[*F* ^2^ > 2σ(*F* ^2^)] = 0.052
*wR*(*F* ^2^) = 0.151
*S* = 1.04
3951 reflections
224 parameters
1 restraint
H atoms treated by a mixture of independent and constrained refinement
Δρ_max_ = 0.49 e Å^−3^
Δρ_min_ = −0.77 e Å^−3^

### 

Data collection: *APEX2* (Bruker, 2009 ▶); cell refinement: *SAINT* (Bruker, 2009 ▶); data reduction: *SAINT*; program(s) used to solve structure: *SHELXS97* (Sheldrick, 2008 ▶); program(s) used to refine structure: *SHELXL97* (Sheldrick, 2008 ▶); molecular graphics: *ORTEP-3* (Farrugia, 1997 ▶) and *DIAMOND* (Brandenburg, 2006 ▶); software used to prepare material for publication: *publCIF* (Westrip, 2010 ▶).

## Supplementary Material

Crystal structure: contains datablocks global, I. DOI: 10.1107/S1600536810030187/hb5589sup1.cif

Structure factors: contains datablocks I. DOI: 10.1107/S1600536810030187/hb5589Isup2.hkl

Additional supplementary materials:  crystallographic information; 3D view; checkCIF report

## Figures and Tables

**Table 1:** Selected bond lengths (Å)

Cu1—O3^i^	1.967 (3)
Cu1—O1^i^	1.975 (3)
Cu1—O2	1.983 (3)
Cu1—O4	1.984 (3)
Cu1—N1	2.220 (3)
Cu1—Cu1^i^	2.6431 (10)

Symmetry code: (i) 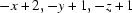.

**Table 2:** Hydrogen-bond geometry (Å, °) *Cg*1 is the centroid of the N1,C5–C9 ring.

*D*—H⋯*A*	*D*—H	H⋯*A*	*D*⋯*A*	*D*—H⋯*A*
N2—H2⋯O4	0.85 (4)	2.30 (2)	3.101 (5)	156 (4)
C4—H4a⋯*Cg*1^ii^	0.96	2.83	3.650 (5)	144

Symmetry code: (ii) 
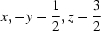
.

**Fig. 1.**
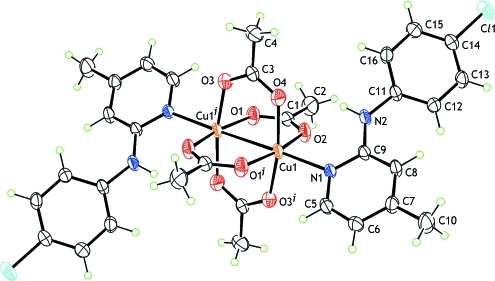
The molecular structure of (I) showing displacement ellipsoids at the 35% probability level. Symmetry code: (i) 2–x, 1–y, 1–z.

**Fig. 2.**
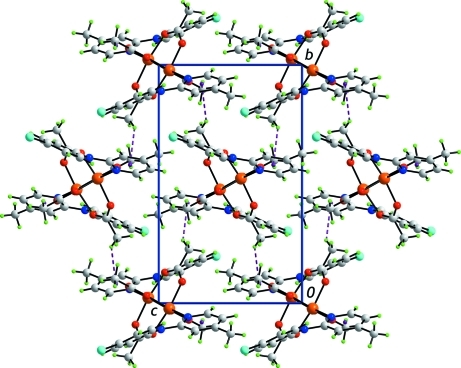
Unit-cell contents shown in projection down the a axis in (I). The C–H···π contacts are shown as purple dashed lines.

## supplementary crystallographic information

### Comment

The binuclear title complex, (I), was studied in connection with the structural characterization of tetrakisacetatobis[(substituted 2-aminopyridyl)copper] complexes, see: Barquín *et al.*, 2004; Seco *et al.*, 2004; Sieroń, 2004; Fairuz *et al.*, 2009). The binuclear copper(II) complex, Fig. 1, is situated about a centre of inversion and features two Cu atoms bridged by four acetate groups. The Cu–O bond distances lie in the experimentally equivalent range 1.967 (3) to 1.984 (3) Å, Table 1. The coordination environment for each Cu atom is completed by a N atom derived from the *N*-4-chloroanilino-4-picoline ligand and the second Cu atom [Cu···Cu^i^ = 2.6431 (10) Å]. The resulting hexa-coordinated geometry is based on an octahedron. An intramolecular N1–H···O4 interaction is noted, Table 2. The *N*-heterocycle is non-planar with the dihedral angle formed between the pyridine and benzene rings being 33.9 (2) °. The major twist in the molecule occurs around the amine group as seen in the value of the C9–N2–C11–C12 torsion angle of 24.1 (8) °. The most obvious intermolecular contact operating in the crystal structure is of the type C–H···π and occurs between methyl-H and pyridine rings, Table 2. These link complex molecules that stack in columns along the *a* axis, Fig. 2.

### Experimental

A mixture of *N*-(4-chlorophenyl)-4-methylpyridin-2-amine (0.2408 g, 1.1 mmol) in acetonitrile (15 ml) and trimethyl orthoformate (10 ml) was heated to 328 K. Copper acetate (0.1 g, 0.5 mmol) dissolved in acetonitrile (15 ml) was added drop wise to the ligand solution. The green solution was left at room temperature and green plates of (I) were collected after a few days.

### Refinement

Carbon-bound H-atoms were placed in calculated positions (C—H 0.93 to 0.96 Å) and were included in the refinement in the riding model approximation, with *U*_iso_(H) set to 1.2 to 1.5*U*_equiv_(C). The N-bound H-atom was located in a difference Fourier map, and was refined with a distance restraint of N–H 0.86±0.01 Å; the *U_iso_* value was freely refined

### Crystal data

**Table:**

[Cu_2_(C_2_H_3_O_2_)_4_(C_12_H_11_ClN_2_)_2_]	*F*(000) = 820
*M**_r_* = 800.61	*D*_x_ = 1.544 Mg m^−^^3^
Monoclinic, *P*2_1_/*c*	Mo *K*α radiation, λ = 0.71073 Å
Hall symbol: -P 2ybc	Cell parameters from 2098 reflections
*a* = 11.7430 (17) Å	θ = 2.5–23.4°
*b* = 15.619 (2) Å	µ = 1.45 mm^−^^1^
*c* = 9.9866 (14) Å	*T* = 296 K
β = 109.901 (2)°	Plate, green
*V* = 1722.3 (4) Å^3^	0.35 × 0.25 × 0.05 mm
*Z* = 2	

### Data collection

**Table:**

Bruker SMART APEX CCD diffractometer	3951 independent reflections
Radiation source: fine-focus sealed tube	2460 reflections with *I* > 2σ(*I*)
graphite	*R*_int_ = 0.065
ω scan	θ_max_ = 27.5°, θ_min_ = 1.8°
Absorption correction: multi-scan (*SADABS*; Sheldrick, 1996)	*h* = −15→13
*T*_min_ = 0.632, *T*_max_ = 0.931	*k* = −19→20
11432 measured reflections	*l* = −11→12

### Refinement

**Table:**

Refinement on *F*^2^	Primary atom site location: structure-invariant direct methods
Least-squares matrix: full	Secondary atom site location: difference Fourier map
*R*[*F*^2^ > 2σ(*F*^2^)] = 0.052	Hydrogen site location: inferred from neighbouring sites
*wR*(*F*^2^) = 0.151	H atoms treated by a mixture of independent and constrained refinement
*S* = 1.04	*w* = 1/[σ^2^(*F*_o_^2^) + (0.064*P*)^2^ + 1.063*P*] where *P* = (*F*_o_^2^ + 2*F*_c_^2^)/3
3951 reflections	(Δ/σ)_max_ = 0.001
224 parameters	Δρ_max_ = 0.49 e Å^−^^3^
1 restraint	Δρ_min_ = −0.77 e Å^−^^3^

### Fractional atomic coordinates and isotropic or equivalent isotropic displacement parameters (Å^2^)

**Table:**

	*x*	*y*	*z*	*U*_iso_*/*U*_eq_	
Cu1	0.92999 (4)	0.47727 (4)	0.57325 (5)	0.03790 (19)	
Cl1	0.17644 (11)	0.30281 (12)	0.10013 (15)	0.0756 (5)	
N1	0.8173 (3)	0.4410 (2)	0.7033 (3)	0.0359 (8)	
N2	0.6604 (3)	0.3905 (3)	0.5143 (4)	0.0514 (11)	
H2	0.712 (3)	0.386 (3)	0.472 (4)	0.054 (14)*	
O1	0.9185 (3)	0.5697 (2)	0.2909 (3)	0.0520 (8)	
O2	0.7991 (2)	0.5312 (2)	0.4143 (3)	0.0526 (9)	
O3	1.0247 (3)	0.4097 (2)	0.3375 (3)	0.0502 (8)	
O4	0.9027 (3)	0.3711 (2)	0.4573 (4)	0.0539 (9)	
C1	0.8177 (4)	0.5643 (3)	0.3087 (5)	0.0424 (10)	
C2	0.7092 (4)	0.6009 (4)	0.1941 (5)	0.0656 (15)	
H2A	0.6375	0.5713	0.1939	0.098*	
H2B	0.7191	0.5942	0.1032	0.098*	
H2C	0.7018	0.6607	0.2122	0.098*	
C3	0.9545 (3)	0.3572 (3)	0.3661 (4)	0.0408 (10)	
C4	0.9312 (4)	0.2732 (3)	0.2895 (5)	0.0569 (13)	
H4A	0.8869	0.2365	0.3312	0.085*	
H4B	1.0070	0.2468	0.2971	0.085*	
H4C	0.8849	0.2825	0.1909	0.085*	
C5	0.8690 (4)	0.4552 (3)	0.8442 (5)	0.0490 (12)	
H5	0.9457	0.4798	0.8764	0.059*	
C6	0.8160 (4)	0.4360 (4)	0.9428 (5)	0.0531 (13)	
H6	0.8555	0.4474	1.0388	0.064*	
C7	0.7010 (4)	0.3986 (3)	0.8961 (4)	0.0456 (11)	
C8	0.6464 (3)	0.3833 (3)	0.7531 (4)	0.0417 (11)	
H8	0.5700	0.3584	0.7193	0.050*	
C9	0.7059 (3)	0.4054 (3)	0.6584 (4)	0.0350 (9)	
C10	0.6402 (5)	0.3728 (4)	1.0011 (5)	0.0710 (17)	
H10A	0.5833	0.4162	1.0044	0.107*	
H10B	0.7002	0.3662	1.0939	0.107*	
H10C	0.5984	0.3195	0.9718	0.107*	
C11	0.5429 (3)	0.3694 (3)	0.4227 (4)	0.0375 (10)	
C12	0.4356 (4)	0.3881 (3)	0.4500 (4)	0.0414 (10)	
H12	0.4400	0.4149	0.5347	0.050*	
C13	0.3230 (4)	0.3668 (3)	0.3517 (5)	0.0447 (11)	
H13	0.2526	0.3776	0.3717	0.054*	
C14	0.3172 (4)	0.3294 (3)	0.2243 (5)	0.0436 (11)	
C15	0.4220 (4)	0.3123 (3)	0.1936 (5)	0.0500 (12)	
H15	0.4171	0.2877	0.1070	0.060*	
C16	0.5334 (4)	0.3321 (3)	0.2928 (5)	0.0474 (12)	
H16	0.6034	0.3202	0.2725	0.057*	

### Atomic displacement parameters (Å^2^)

**Table:**

	*U*^11^	*U*^22^	*U*^33^	*U*^12^	*U*^13^	*U*^23^
Cu1	0.0227 (2)	0.0514 (4)	0.0388 (3)	−0.0041 (2)	0.00946 (19)	0.0002 (3)
Cl1	0.0348 (6)	0.1143 (13)	0.0618 (8)	−0.0131 (7)	−0.0044 (5)	−0.0148 (8)
N1	0.0246 (15)	0.050 (2)	0.0316 (17)	−0.0048 (15)	0.0081 (13)	−0.0007 (16)
N2	0.0275 (17)	0.091 (3)	0.037 (2)	−0.021 (2)	0.0132 (15)	−0.011 (2)
O1	0.0338 (15)	0.075 (2)	0.0466 (18)	0.0082 (16)	0.0124 (14)	0.0134 (17)
O2	0.0260 (14)	0.080 (3)	0.0495 (18)	0.0036 (15)	0.0100 (13)	0.0128 (17)
O3	0.0449 (17)	0.058 (2)	0.0533 (19)	−0.0118 (16)	0.0234 (15)	−0.0082 (16)
O4	0.0485 (18)	0.058 (2)	0.064 (2)	−0.0131 (16)	0.0299 (17)	−0.0089 (17)
C1	0.031 (2)	0.049 (3)	0.043 (2)	0.001 (2)	0.0071 (18)	−0.005 (2)
C2	0.043 (3)	0.085 (4)	0.056 (3)	0.023 (3)	0.002 (2)	0.010 (3)
C3	0.0264 (18)	0.050 (3)	0.041 (2)	−0.0003 (19)	0.0048 (17)	−0.003 (2)
C4	0.053 (3)	0.052 (3)	0.061 (3)	−0.012 (2)	0.013 (2)	−0.008 (3)
C5	0.033 (2)	0.074 (4)	0.039 (2)	−0.017 (2)	0.0113 (18)	−0.007 (2)
C6	0.042 (2)	0.081 (4)	0.036 (2)	−0.017 (3)	0.0125 (19)	−0.011 (2)
C7	0.033 (2)	0.069 (3)	0.036 (2)	−0.004 (2)	0.0133 (17)	0.005 (2)
C8	0.0242 (18)	0.059 (3)	0.041 (2)	−0.0074 (19)	0.0093 (17)	0.006 (2)
C9	0.0247 (17)	0.043 (3)	0.033 (2)	−0.0035 (17)	0.0049 (15)	0.0015 (18)
C10	0.051 (3)	0.121 (5)	0.045 (3)	−0.016 (3)	0.021 (2)	0.005 (3)
C11	0.0256 (18)	0.049 (3)	0.035 (2)	−0.0080 (18)	0.0068 (16)	0.0046 (19)
C12	0.033 (2)	0.054 (3)	0.037 (2)	−0.002 (2)	0.0110 (18)	−0.004 (2)
C13	0.0257 (19)	0.056 (3)	0.050 (3)	0.005 (2)	0.0102 (18)	0.003 (2)
C14	0.030 (2)	0.051 (3)	0.044 (2)	−0.004 (2)	0.0039 (17)	−0.001 (2)
C15	0.039 (2)	0.065 (3)	0.041 (2)	0.000 (2)	0.0069 (19)	−0.006 (2)
C16	0.030 (2)	0.073 (4)	0.040 (2)	−0.004 (2)	0.0140 (18)	−0.001 (2)

### Geometric parameters (Å, °)

**Table:**

Cu1—O3^i^	1.967 (3)	C4—H4B	0.9600
Cu1—O1^i^	1.975 (3)	C4—H4C	0.9600
Cu1—O2	1.983 (3)	C5—C6	1.366 (6)
Cu1—O4	1.984 (3)	C5—H5	0.9300
Cu1—N1	2.220 (3)	C6—C7	1.397 (6)
Cu1—Cu1^i^	2.6431 (10)	C6—H6	0.9300
Cl1—C14	1.745 (4)	C7—C8	1.373 (6)
N1—C9	1.350 (5)	C7—C10	1.511 (6)
N1—C5	1.348 (5)	C8—C9	1.397 (5)
N2—C9	1.373 (5)	C8—H8	0.9300
N2—C11	1.411 (5)	C10—H10A	0.9600
N2—H2	0.85 (4)	C10—H10B	0.9600
O1—C1	1.258 (5)	C10—H10C	0.9600
O1—Cu1^i^	1.975 (3)	C11—C16	1.392 (6)
O2—C1	1.259 (5)	C11—C12	1.407 (6)
O3—C3	1.262 (5)	C12—C13	1.393 (5)
O3—Cu1^i^	1.967 (3)	C12—H12	0.9300
O4—C3	1.274 (5)	C13—C14	1.380 (6)
C1—C2	1.506 (6)	C13—H13	0.9300
C2—H2A	0.9600	C14—C15	1.392 (6)
C2—H2B	0.9600	C15—C16	1.381 (6)
C2—H2C	0.9600	C15—H15	0.9300
C3—C4	1.497 (6)	C16—H16	0.9300
C4—H4A	0.9600		
			
O3^i^—Cu1—O1^i^	88.89 (14)	H4A—C4—H4C	109.5
O3^i^—Cu1—O2	89.88 (14)	H4B—C4—H4C	109.5
O1^i^—Cu1—O2	168.34 (12)	N1—C5—C6	124.2 (4)
O3^i^—Cu1—O4	168.17 (12)	N1—C5—H5	117.9
O1^i^—Cu1—O4	91.08 (15)	C6—C5—H5	117.9
O2—Cu1—O4	87.76 (14)	C5—C6—C7	118.5 (4)
O3^i^—Cu1—N1	95.02 (13)	C5—C6—H6	120.7
O1^i^—Cu1—N1	94.59 (12)	C7—C6—H6	120.7
O2—Cu1—N1	97.06 (12)	C8—C7—C6	118.4 (4)
O4—Cu1—N1	96.77 (13)	C8—C7—C10	120.8 (4)
O3^i^—Cu1—Cu1^i^	83.47 (9)	C6—C7—C10	120.8 (4)
O1^i^—Cu1—Cu1^i^	84.00 (9)	C7—C8—C9	119.8 (4)
O2—Cu1—Cu1^i^	84.35 (9)	C7—C8—H8	120.1
O4—Cu1—Cu1^i^	84.76 (9)	C9—C8—H8	120.1
N1—Cu1—Cu1^i^	177.94 (9)	N1—C9—N2	113.9 (3)
C9—N1—C5	117.2 (3)	N1—C9—C8	121.9 (3)
C9—N1—Cu1	127.9 (3)	N2—C9—C8	124.2 (4)
C5—N1—Cu1	115.0 (3)	C7—C10—H10A	109.5
C9—N2—C11	131.6 (3)	C7—C10—H10B	109.5
C9—N2—H2	116 (3)	H10A—C10—H10B	109.5
C11—N2—H2	111 (3)	C7—C10—H10C	109.5
C1—O1—Cu1^i^	123.3 (3)	H10A—C10—H10C	109.5
C1—O2—Cu1	122.5 (3)	H10B—C10—H10C	109.5
C3—O3—Cu1^i^	125.2 (3)	C16—C11—N2	116.9 (4)
C3—O4—Cu1	122.5 (3)	C16—C11—C12	118.3 (4)
O1—C1—O2	125.9 (4)	N2—C11—C12	124.6 (4)
O1—C1—C2	117.4 (4)	C13—C12—C11	120.8 (4)
O2—C1—C2	116.7 (4)	C13—C12—H12	119.6
C1—C2—H2A	109.5	C11—C12—H12	119.6
C1—C2—H2B	109.5	C14—C13—C12	119.2 (4)
H2A—C2—H2B	109.5	C14—C13—H13	120.4
C1—C2—H2C	109.5	C12—C13—H13	120.4
H2A—C2—H2C	109.5	C13—C14—C15	120.9 (4)
H2B—C2—H2C	109.5	C13—C14—Cl1	119.6 (3)
O3—C3—O4	123.9 (4)	C15—C14—Cl1	119.5 (3)
O3—C3—C4	118.2 (4)	C16—C15—C14	119.5 (4)
O4—C3—C4	117.8 (4)	C16—C15—H15	120.3
C3—C4—H4A	109.5	C14—C15—H15	120.3
C3—C4—H4B	109.5	C15—C16—C11	121.2 (4)
H4A—C4—H4B	109.5	C15—C16—H16	119.4
C3—C4—H4C	109.5	C11—C16—H16	119.4
			
O3^i^—Cu1—N1—C9	134.2 (4)	Cu1—N1—C5—C6	−178.7 (4)
O1^i^—Cu1—N1—C9	−136.5 (4)	N1—C5—C6—C7	0.3 (8)
O2—Cu1—N1—C9	43.7 (4)	C5—C6—C7—C8	−0.3 (8)
O4—Cu1—N1—C9	−44.9 (4)	C5—C6—C7—C10	177.6 (5)
O3^i^—Cu1—N1—C5	−47.1 (3)	C6—C7—C8—C9	−0.2 (7)
O1^i^—Cu1—N1—C5	42.2 (3)	C10—C7—C8—C9	−178.1 (5)
O2—Cu1—N1—C5	−137.6 (3)	C5—N1—C9—N2	−178.3 (4)
O4—Cu1—N1—C5	133.8 (3)	Cu1—N1—C9—N2	0.4 (6)
O3^i^—Cu1—O2—C1	84.2 (4)	C5—N1—C9—C8	−0.6 (6)
O1^i^—Cu1—O2—C1	0.3 (9)	Cu1—N1—C9—C8	178.0 (3)
O4—Cu1—O2—C1	−84.2 (4)	C11—N2—C9—N1	−167.2 (5)
N1—Cu1—O2—C1	179.2 (4)	C11—N2—C9—C8	15.3 (8)
Cu1^i^—Cu1—O2—C1	0.8 (4)	C7—C8—C9—N1	0.7 (7)
O3^i^—Cu1—O4—C3	7.9 (9)	C7—C8—C9—N2	178.1 (5)
O1^i^—Cu1—O4—C3	−81.8 (3)	C9—N2—C11—C16	−159.9 (5)
O2—Cu1—O4—C3	86.6 (3)	C9—N2—C11—C12	24.1 (8)
N1—Cu1—O4—C3	−176.6 (3)	C16—C11—C12—C13	2.3 (7)
Cu1^i^—Cu1—O4—C3	2.0 (3)	N2—C11—C12—C13	178.3 (4)
Cu1^i^—O1—C1—O2	2.0 (7)	C11—C12—C13—C14	−2.1 (7)
Cu1^i^—O1—C1—C2	−178.3 (3)	C12—C13—C14—C15	0.5 (7)
Cu1—O2—C1—O1	−1.8 (7)	C12—C13—C14—Cl1	−179.8 (4)
Cu1—O2—C1—C2	178.5 (3)	C13—C14—C15—C16	0.8 (8)
Cu1^i^—O3—C3—O4	0.4 (6)	Cl1—C14—C15—C16	−178.9 (4)
Cu1^i^—O3—C3—C4	−179.1 (3)	C14—C15—C16—C11	−0.5 (8)
Cu1—O4—C3—O3	−2.1 (6)	N2—C11—C16—C15	−177.3 (4)
Cu1—O4—C3—C4	177.4 (3)	C12—C11—C16—C15	−1.0 (7)
C9—N1—C5—C6	0.1 (7)		

Symmetry codes: (i) −*x*+2, −*y*+1, −*z*+1.

### Hydrogen-bond geometry (Å, °)

**Table:**

Cg1 is the centroid of the N1,C5–C9 ring.

**Table:**

*D*—H···*A*	*D*—H	H···*A*	*D*···*A*	*D*—H···*A*
N2—H2···O4	0.85 (4)	2.30 (2)	3.101 (5)	156 (4)
C4—H4a···Cg1^ii^	0.96	2.83	3.650 (5)	144

Symmetry codes: (ii) *x*, −*y*−1/2, *z*−3/2.
